# Carbon Footprint of Telemedicine Solutions - Unexplored Opportunity for Reducing Carbon Emissions in the Health Sector

**DOI:** 10.1371/journal.pone.0105040

**Published:** 2014-09-04

**Authors:** Åsa Holmner, Kristie L. Ebi, Lutfan Lazuardi, Maria Nilsson

**Affiliations:** 1 Department of Radiation Sciences, Umeå University, Umeå, Sweden; 2 ClimAdapt, LLC, Seattle, Washington, United States of America; 3 Department of Public Health, Faculty of Medicine, Gadjah Mada University, Yogyakarta, Indonesia; 4 Department of public health and clinical medicine, epidemiology and global health, Umeå University, Umeå, Sweden; US Army Engineer Research and Development Center, United States of America

## Abstract

**Background:**

The healthcare sector is a significant contributor to global carbon emissions, in part due to extensive travelling by patients and health workers.

**Objectives:**

To evaluate the potential of telemedicine services based on videoconferencing technology to reduce travelling and thus carbon emissions in the healthcare sector.

**Methods:**

A life cycle inventory was performed to evaluate the carbon reduction potential of telemedicine activities beyond a reduction in travel related emissions. The study included two rehabilitation units at Umeå University Hospital in Sweden. Carbon emissions generated during telemedicine appointments were compared with care-as-usual scenarios. Upper and lower bound emissions scenarios were created based on different teleconferencing solutions and thresholds for when telemedicine becomes favorable were estimated. Sensitivity analyses were performed to pinpoint the most important contributors to emissions for different set-ups and use cases.

**Results:**

Replacing physical visits with telemedicine appointments resulted in a significant 40–70 times decrease in carbon emissions. Factors such as meeting duration, bandwidth and use rates influence emissions to various extents. According to the lower bound scenario, telemedicine becomes a greener choice at a distance of a few kilometers when the alternative is transport by car.

**Conclusions:**

Telemedicine is a potent carbon reduction strategy in the health sector. But to contribute significantly to climate change mitigation, a paradigm shift might be required where telemedicine is regarded as an essential component of ordinary health care activities and not only considered to be a service to the few who lack access to care due to geography, isolation or other constraints.

## Introduction

The health care sector is facing a series of major challenges. In developed countries, the health care sector is committed to improving the provision of care, while at the same reducing costs. In developing countries, the health sector is facing the challenge of meeting the fundamental right of all citizens to adequate health according to the WHO and UN goals of universal health coverage and healthy life expectancy. Challenges range from aging populations with an increasing prevalence of lifestyle related disease, to changing disease patterns because of development patterns and global environmental changes [1], [2]. At the same time, the health care sector contributes significantly to climate change [3], [4], which means that if the sector does not introduce climate friendly policies and practices, it paradoxically will continue to contribute directly and indirectly to negative health impacts through its emissions of CO_2_ and other greenhouse gases. To meet the growing demand of health care resources without furthering climate change, future health services must be built on sustainable and low-carbon systems and work models.

Information and communication technology (ICT) has been suggested as one solution for reducing the carbon footprint of many sectors, including the health care sector [5]. Telemedicine, which is defined as the use of ICT to provide health services across distance, time, or other barriers, has been suggested to be a potent tool to reduce emissions from travel that, according to the UK National Health Services (NHS), represent as much as 18% of the total carbon footprint of the UK health sector [4]. Telemedicine covers a broad range of technologies and activities that have been applied to many different clinical disciplines, such as radiology, pathology, dermatology, rehabilitation, and chronic disease management. Large-scale clinical studies are often lacking, but in general telemedicine studies show high patient satisfaction and acceptance [6], [7], although evidence on cost effectiveness is still relatively weak [8]. Regarding clinical outcomes, some telemedicine applications, such as specialist rehabilitation using videoconferencing, have shown to be comparable to or even more efficient than traditional interventions [9]. In our experience, this can be explained by the increased access to specialists that makes it possible to commit to a more intense rehabilitation regime that would have been feasible if the patient had been required to travel to the clinic for each appointment.

Attempts have been made to evaluate telemedicine programs from a climate mitigation perspective by estimating the potential reduction in tailpipe emissions [10]–[14]. However, to disregard the impacts from the technology used to facilitate telemedicine can be seriously misleading and it is important to take into account the cost of manufacturing, using and discarding the equipment, to evaluate the actual impact of different virtual meeting solutions [15], [16].

To assess the carbon costs and benefits of telemedicine, we performed a simplified life cycle inventory for telemedicine activities within two rehabilitation units at the Umeå University Hospital in Northern Sweden. These two units have provided evidence that rehabilitation using telemedicine, i.e., telerehabilitation, is a cost efficient and well-accepted alternative to traditional care in Northern Sweden (personal communication). Net emissions for the telemedicine appointments in these two cases were compared to care-as-usual scenarios constructed using the patient's place of residence. Thresholds for when the telemedicine work model becomes favorable were estimated and sensitivity analyses performed to provide insight into (1) what factors contribute most to carbon emission in the telemedicine work model, (2) what influence technological set-ups and different use scenarios have on net carbon emission and (3) what would be the impact of greener transport options on the carbon reduction potential. These are the first such assessments of telemedicine activities and have the potential to contribute important information for the development of telemedicine guidelines in sustainable health care practice.

## Methods

### Ethics statement

This study is based on data from two clinical units within the University Hospital of Umeå. The study is based on aggregated data and patient information used in the study was anonymized and de-identified prior to analysis. It was thus judged by the Regional Ethical Review Board in Umeå that no ethical approval was needed.

### Data capture

To assess the carbon footprint of telerehabilitation at the University Hospital of Northern Sweden we analyzed data from two units offering specialist rehabilitation using telemedicine. These are the rehabilitation unit of the hand and plastic surgery section, which is part of the centre for reconstructive surgery, and the speech therapy clinic, which is a county clinic belonging to the ear, nose and throat clinic. The reason for including these two clinics in our study was twofold; telemedicine has become a well integrated part of the clinical activities in these units and there are extensive records of their telemedicine appointments available for analysis. Telemedicine appointments were compared with care-as-usual scenarios that require the patient travel to the hospital for a face-to-face visit. Patients enrolled in the telemedicine programs are primarily inhabitants of Västerbotten County, but the hospital also provides specialized health care to the northern care region (approximately the northern half of Sweden), as well as to some patients from other counties. Travel distances are estimated as the distance from the town closest to the patient's place of residence to Umeå where the university hospital is located. One-way distances vary from less than one km to 700 km although the hand rehabilitation unit only registers travel distances above 2.5 km (one way). Car is the transport option assessed in our scenarios as most patients, particularly in the speech therapy clinic, utilize car or subsidized taxi services to reach the hospital. Patients from other counties may travel by other means, such as airplane or bus, but this is not accounted for in our study. All calculations were performed using Microsoft Excel.

### Hand- and plastic surgery section

The study included all patients involved in the telerehabilitation program from January to December 2012. Appointments included follow-ups, interventions, consultations, and assessments of various conditions, such as amputations of one or more fingers, osteoarthritis, flexor tendon injuries, radius fractures, finger fractures, and ligament injuries. There were 238 telemedicine appointments during this period that thus avoided travel to Umea. Of these, 81 were conducted in the patient's home using a PC or tablet computer and 157 at the closest primary health centre using standard videoconferencing equipment. Travel to appointments made at the primary health centre is not accounted for. Based on the patient's places of residence, an accumulated travel distance of 82,310 km was avoided during the study. Data on the exact length of individual appointments are lacking, but they typically varied from 10 to 50 minutes with an average of 25 minutes (verbal communication).

### Speech therapy unit

The study included data for all patients involved in a telemedicine project conducted in 2005–2006 at the speech therapy unit. Today, telemedicine is well integrated into the clinical activities; however, readily accessible statistics on the patients' place of residence (used to calculate travel-related emissions for comparison) was only available from the time of the project. Telemedicine treatment was delivered to patients of all ages for conditions including aphasia, dysarthria, and dyslexia. 481 therapy sessions using telemedicine were performed either in the patients' home or at the closest primary health centre, although details about exact location were not available. Data on the length of individual appointments were not available but varied from 30 to 40 minutes, with an average of 35 minutes (verbal communication). Based on the patient's place of residence, an accumulated travel distance of 154,842 km was avoided during the study.

### Life cycle assessment

A Life Cycle Assessment (LCA) is defined as a complete ecological assessment of all energy, material, and waste flows of a product, and their impact on the environment. This “cradle to grave” evaluation begins with the design of the product and ends with the disposal or recycling of material and components (end-of-life). Typically, LCAs are performed in several steps, starting with a life cycle inventory to determine the raw materials and energy used and the emissions that occur during the life cycle of a product. This inventory is followed by a life cycle impact assessment to estimate the impacts of these emissions and raw material depletions on, for example, human health or certain ecosystems. If the focus of the study is on a single waste product it can be specified as a stream lined LCA.

This study adopts the form of a simplified, stream lined life cycle inventory, with the aim to evaluate the most important aspects of telemedicine with respect to CO_2_ emissions. We chose one hour as the functional unit in the telemedicine scenario. This is a typical meeting duration and is, in addition, a convenient entity to work with considering that the units W and kWh are applied in our calculations.

**Figure 1:**
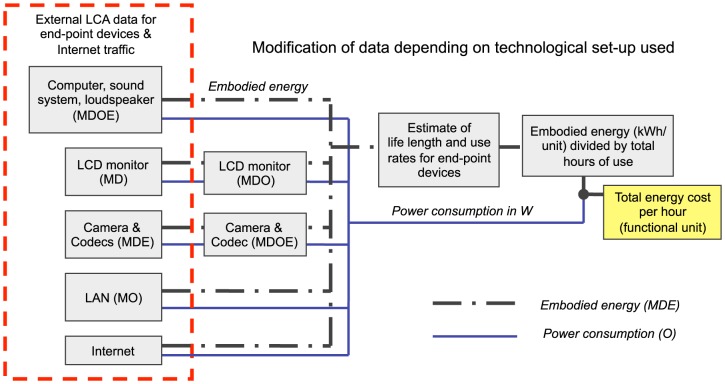
Methods summary. The study is based on the method used by Ong et al. that builds on existing LCA data for end-point devices used in videoconferencing and emission estimates for Internet traffic [17]. Completeness of the LCA data varies between devices, but includes energy costs and/or carbon emissions generated during manufacturing (M), distribution (D), operation (O) and end-of-life stages (E). Emissions data for MDE are provided in, or has been converted to, energy equivalents (kWh/unit) and is called embodied energy. Data for the videoconferencing solution (monitors, camera and video codecs) were modified to better fit our technological set-up. To obtain the hourly carbon cost of telerehabilitation in kgCO_2_e, we divided the embodied energy with estimates of the life length and use rates of all equipment, and applied a conversion factor of 0.6 kg CO_2_e/kWh [17], [20]. See also Equation 1.

The study builds on results from life cycle inventories on hardware and software required to connect the patient and the specialist, called mediated meeting solutions or videoconferencing solutions. We largely adopted the strategy described in Ong et al. [17] that takes into account end-point devices, such as computers [18], monitors, cameras, local area network (LAN) components [19] and video codecs used to compress and decompress digital video signals, as well as the costs for Internet traffic [19], although technically, data is transmitted using the dedicated hospital network, Sjunet. The method was modified to fit our device set-ups (see Figure 1 and Table 1) and assumptions on life length and use rates of the equipment. To make the protocol more generic, life cycle and operating carbon costs are given in kWh to account for the carbon footprint of energy production itself, which may differ significantly between countries and different stages of the product life cycle.

**Table 1:** Life cycle and operating costs of end point devices.

	*Power consumption (W)*	*Embodied energy (kWh/unit)* ∧	*LCA phases included* *
**PC**			
Desktop	150	583	M D O E
Laptop	40	378	M D O E
Camera	9.5	33	M D O E
Sound system	4.1	104	M O
Microphone	2.5	52	M O
**Monitor**			
46” NEC LCD	188	145¤	M D O
**Videoconference**			
Camera + codec (Cisco SX20)	40	134#	M D O E
Local Area Network (LAN) end-points	20	278	M O

* M = manufacturing, D = distribution, O = operation, E = end-of-life (disposal & recycle)

∧ In the original reference, some values were converted from CO_2_e to MJ using a conversion factor of 0.6 kg CO_2_e/kWh [17], [20]. Data were further converted from MJ to kWh to better fit our calculations.

# Embodied energy of the Cisco SX20 camera and codec was estimated from the embodied energy of camera and entry level video codec used in Ong et al. [17].

¤ Data on the embodied energy of the NEC LCD monitor was based on an active screen size of 1018×572.7 mm and calculated from data provided by Ong et al. [17].

The total carbon cost of a virtual meeting was estimated by adding i) the cumulative emissions generated by all equipment during the use-phase (from energy consumption) and ii) emissions generated during design, manufacturing, disposal and recycling of the equipment, whenever these data were available. To obtain an hourly carbon cost of a mediated meeting (functional unit), the cost of operating the equipment for one hour was added to the carbon emissions generated throughout all other life cycle phases amortized over the whole life length of the equipment. In energy terms this can be expressed as; 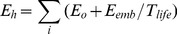
(1)where E_h_ is the total hourly energy cost in kWh (functional unit) for a videoconferencing set-up consisting of a specific combination of end-point devices or set of devices (i). E_o_ is the hourly energy consumption of the end-point device during operation, E_emb_ is the accumulated embodied energy for all other life cycle phases available (see Table 1) and T_life_ is the expected number of hours in use. A generalized conversion factor of 0.6 kCO_2_e/kWh was used to convert these results to carbon dioxide equivalents, based on the methods developed by Ong and Malmodin [17], [20], because the processes of designing, manufacturing, using, and disposing of the technology may differ across locations and countries. It is thus likely that we overestimate the carbon cost of the use phase because northern Sweden has access to environmentally friendly electricity in the form of hydropower and wind power. Life cycle data on computers, monitors, and other end-point devices are summarized in Table 1. Data on the Internet operating expenditures (opex) and embodied energy for different bandwidths are summarized in Table 2.

**Table 2:** Estimates of the Internet opex and embodied energy.

	Maximum estimates *	
Bandwidth	Networking energy opex (kWh)	Embodied energy cost (kW)
20 Mbit/s	32	29.3
10 Mbit/s	16	14.6
4 Mbit/s	6.4	5.6
2 Mbit/s	3.2	2.9
1 Mbit/s	1.6	2.2
768 kbit/s	1.2	1.5
512 kbit/s	0.8	1.1

*For simplicity and to avoid underestimating the cost of Internet traffic, the network operating expenditure (opex) and embodied energy (in kWh) are based on the maximum estimate of the operating energy intensity (3.61 kWh/GB) and embodied energy (3.33 kWh/GB) of the Internet. To calculate the opex in kWh, the bandwidth was converted to bytes per hour (or MB/h).

To enable comparison with emissions generated during a traditional physical meeting, we accounted for the life cycle carbon costs of travelling. The estimates were primarily based on the research by Lenzen et al. [21] that addresses tailpipe emissions as well as energy consumption and/or carbon emissions generated during other life cycle phases of transport, such as manufacturing of cars, fuels, and road infrastructure. According to Lenzen, the total carbon cost of a car is 0.86 kgCO_2_e/km. To account for improved energy efficiency in more modern set-ups, we assessed more recent data by Leduc et al. [22] that accounts for life cycle phases related to automobiles and fuels, exclusively. This study generated a total of 0.25–0.27 kgCO_2_e/km for the most commonly purchased petrol and diesel cars in Europe in 2010; we utilized the value of 0.26 kgCO_2_e/km in our study. The EU emissions target of 0.130 kg CO_2_ for automobiles in 2015 is also addressed in the sensitivity analysis.

### System identification

Telemedicine appointments were conducted with different technological set-ups depending on location of the treatment; at home or at the closest primary health centre. For home treatments, the patient and the specialist are both assumed to be using a standard desktop PC with additional web camera, microphone, and loud speakers. The patient's computer is assumed to be used for 780 hours in total before disposal, based on a use rate of 5 hours per week for three years. This is a very rough estimate because the users vary significantly in age and computer habit; therefore, use rates are further addressed in the sensitivity analysis. The PC of the specialist is used for 7,300 hours before disposal based on an average use rate of 5 hours per day and a life expectancy of four years (data from the investment database). The bandwidth is set to 512 kbps, which is considered to be an acceptable lower limit for treatment provided in the home.

For treatment provided in the primary health centre, the patient is assumed to be utilizing a Cisco TelePresence SX20 with a 46" LCD monitor from NEC (or equivalent) that is used for 360 hours before disposal, based on an average use of 60 hours per year in six years. This estimate is derived from the average use-rate of videoconferencing equipment in the county council, which is 120 hours per year, with the number decreased to better fit the rural primary health centers. The specialist is using the same Cisco system but the use rate is assumed to be 240 hours per year for six years on average, i.e., 1440 hours. This estimate is based on the average use rate of videoconferencing equipment, with the number increased to better fit the two clinics as they are more frequent users of telemedicine than the average department. The LAN is expected to be operating for 14,600 hours derived from 10 hours per day for four years. The data transfer rate is estimated as 4Mbps, slightly exceeding the bandwidth typically used for telerehabilitation. The power consumption and embodied energy of computers [18], monitors, and audio/video peripherals are based on literature values for equivalent products, including the LAN end-point devices.

Access to information on the technological set-ups used for individual appointments was only available for the hand and plastic surgery unit. Therefore, we estimated a realistic upper and lower emission limit based on the following scenarios. These scenarios were applied to both clinics.

Upper bound scenario: The upper bound calculations were based on the scenario described above for treatment in the primary health centre, but with the addition of a second 46” screen in the videoconference room of the specialist.

Lower bound scenario: The lower bound calculations were based on the scenario described above for treatments provided in the patients' home; two standard desktop PCs with use rates of 780 hours (patient) and 7,300 hours (specialist) and a bandwidth of 512 kbps. Regarding carbon costs of Internet traffic, the same assumptions apply as for the upper bound scenario to avoid underestimating the carbon costs. The patients home LAN is likely to be of significantly lower complexity than the hospital LAN and both power consumption and embodied energy costs are likely to be significantly lower. Nonetheless, we chose to apply the same estimates as the higher bound scenario, with the only exception that the use rate applied to the patient scenario is 780 hours, similar to the other end-point devices.

## Results

### Carbon cost comparisons

**Figure 2:**
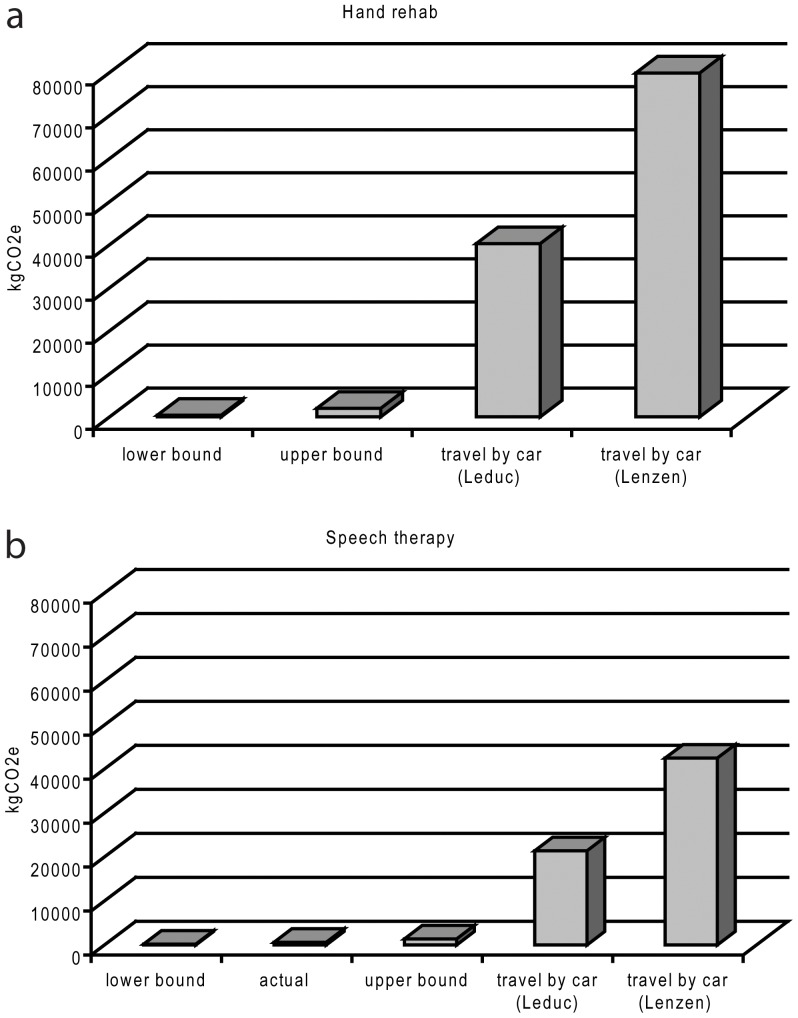
Summary of results. Net emissions for the two scenarios. Hand and plastic surgery section (a), and speech therapy unit (b). Information on the technological set-up used for individual appointments was only available in the hand and plastic surgery section (actual). Therefore, we applied the upper and lower bound scenarios in the speech therapy clinic.

For the hand and plastic surgery clinic, the carbon cost of the 238 telemedicine appointments during 2012 was 602 kgCO_2_e based on our baseline assumptions. This corresponds to an average of 1.4–2.8% of the carbon costs of travelling to/from the clinic by car or subsidized taxi services, based on a total avoided travel distance of 82,310 km for study patients. Based on the upper and lower bound scenarios, a telerehabilitation visit in the hand and plastic surgery clinic generated 0.4–0.9% and 3.2–6.4% of the expected carbon costs for a traditional face-to-face appointment, respectively. Similar numbers were obtained in the speech therapy clinic. In summary, the telerehabilitation activities of the two clinics resulted in a cut in carbon emissions by 15–250 times for the telemedicine work model compared to traditional care. Data on the carbon costs of the different scenarios are summarized in Table 3 and in Figure 2.

**Table 3:** Accumulated life cycle carbon costs of telemedicine versus face-to-face meetings.

	Speech therapy (481 visits)		Hand rehabilitation (238 visits)	
	kWh	kgCO_2_e*	kWh	kgCO_2_e
**Telemedicine**				
Authentic conditions∧			1004	602
Lower bound	741	409	305	183
Upper bound	3307	1984	818	1364
**Face-to-face visit**				
Travel by car (Lenzen)		79 909		42 472
Travel by car (Leduc)		40 258		21 400
**Emissions with respect to car travel (Lenzen/Leduc)**				
Authentic conditions			1.4%/2.8%	
Lower bound	0.6%/1,0%		0.4%/0.9%	
Upper bound	2.5%/4,9%		3.2%/6.4%	

*Based on a conversion factor of 0.6 CO2e/kWh

∧ Based on 157 connections to primary health centres using videoconferencing solutions and 81 connections to the patients home using desktop solutions. Such detailed data were not available in the speech therapy section.

Based on the upper and lower bound scenarios defined in this paper, a one hour telemedicine appointment was estimated to generate 1.86 and 8.43 kgCO_2_e, respectively. Consequently, telerehabilitation is carbon cost-effective once there is a need for the patient to travel at least 3.6 km by car for a one-hour appointment using the Lenzen estimate [21] and 7.2 km based on the Leduc estimate [22]. Corresponding values for the upper bound videoconference scenario are 16 km and 32 km, respectively. In reality, appointments are often shorter than one hour. For the care model described in this paper, these distances may well be reduced by 50%.

### Sensitivity analysis

**Figure 3:**
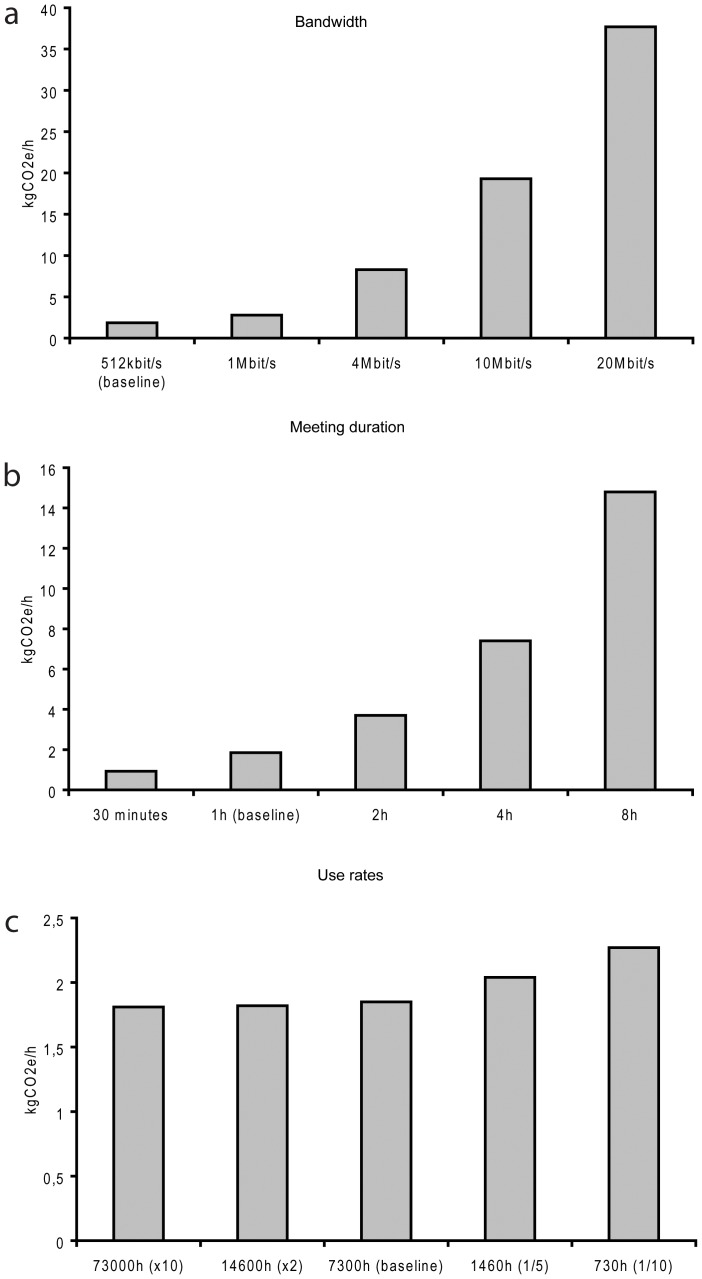
Sensitivity analyses. The analyses are based on the lower bound emissions scenario that emits 1.85 kgCO_2_e during a one hour meeting. All other factors were kept constant while changing the bandwidth (a), meeting duration (b) or use rates (c), respectively.

Sensitivity analyses were performed to gain a better understanding of how different choices in technological set-up and usage of videoconference equipment affect the magnitude of carbon emission for telerehabilitation. Sensitivity analyses were also performed to address LCA data on passenger transport. Results from the sensitivity analyses are summarized below and in Figure 3.

It is clear from the upper and lower bound scenarios that the choice of hardware affects net emission rates. Based on the upper and lower bound scenarios, a one-hour meeting emits 8.43 kgCO_2_e and 1.85 kgCO_2_e, respectively. Thus, a standard desktop solution produces approximately 20% of the carbon equivalents of the Cisco SX20 based on our assumptions. The difference would be even more significant for dedicated desktop videoconferencing solutions, such as the Cisco EX60 that has a significantly lower power consumption in the use phase, or when compared to more complex tele-presence systems or multipart meeting solutions. Meeting duration is another of the dominant factors influencing the carbon emissions of telerehabilitation. A two-hour meeting would essentially produce twice the emission of a one-hour meeting if the use rates are kept constant and within reasonable limits. Another important factor is bandwidth given that data transfer is one of the most energy consuming processes of videoconferencing. For the upper bound scenario, data transfer contributes 87% of the total emissions and the corresponding emissions for the lower bound scenario are approximately 50%. Because the energy consumption of the Internet is calculated in kWh/GB, there is a linear increase in carbon emission with an increase in bandwidth, at least theoretically. Hence, for a one-hour meeting using the otherwise fixed lower bound set-up, the net carbon emissions would increase by more than 200% when increasing the bandwidth from 4 to 10 Mbps. A decrease to 1 Mbps would yield a corresponding 4-times reduction in energy consumption for data transfer and a 3-times reduction in total carbon emissions.

The use rates and expected life of the equipment also will influence the hourly energy and carbon cost of telerehabilitation given that the embodied energy is amortized over the lifetime of the equipment. The use rate of the specialist applied to the lower bound scenario is estimated at 5 hours per day for four years on average, i.e., 7,300 hours in total. A ten-time reduction in use rate would increase the net carbon emission by 20% for a one-hour meeting. However, if the use rates were to increase ten times the corresponding reduction in carbon emission would only be about 2%. Thus, the contribution from the embodied energy to the hourly emission is most influential on equipment used infrequently with the current lower bound estimates.

Finally, the Lenzen [21] reference used to assess the carbon emissions from patient transport is relatively old (1999) and using the newer reference [22] is unrealistic because all cars used are unlikely to be newer models. Moreover, this reference did not account for additional emissions generated by building and maintaining the road infrastructure, for example. Hence, sensitivity analysis was used to address the impact of lower tailpipe emissions and other life cycle contributions to emissions due to utilization of more modern automobiles. If we account for an annual 5% reduction of all life cycle phases of a private car based on an average emission of 0.86 kgCO2e/km according to Lenzen et al., the net emissions of all face-to-face meetings in the hand rehabilitation clinic in 2012 would be 22,950 kgCO_2_e, which still corresponds to 38 times the emissions of the telerehabilitation scenario. If we perform the same calculations using the EU target for automobiles in 2015 of 0.130 kgCO_2_e/km, emissions are reduced to 6,419 kgCO_2_e, which are ten times the carbon emissions with respect to telerehabilitation in the hand rehabilitation clinic. These calculations do not take into account corresponding reductions in life cycle costs of the videoconferencing equipments.

## Discussion

The carbon footprint of telemedicine services has been assessed by estimating the reduction in carbon emission due to reduced need for transportation [10]–[14]. However, the environmental impact of ICT is complex and to look at travel savings alone is misleading[5]. By performing a simplified LCA we accounted for the carbon cost of telemedicine and not just the potential to reduce tailpipe emissions. This is a new contribution to the scientific literature. Our analysis reveals that factors such as choice of teleconferencing solutions, duration of the appointment, capacity of the Internet connection, and use rates of the technology contribute to emissions to various degrees. These results are highly policy relevant. The outcome stresses the benefits of using telemedicine for short meetings and implies that the choice of bandwidth should be based on the clinical need rather than the access to highest possible Internet capacity. At higher bandwidths, data transfer is the main contributor to emissions of telerehabilitation, reaching 87% of total emissions in our upper bound scenario. It is also clear that traditional videoconferencing solutions emit more carbon than desktop solutions per hour of usage, and that use rates should be considered, particularly for equipment used infrequently. Careful planning is thus needed on the local level to make the best use of a videoconference system, which could be a very poor investment if wrongly placed or when in low demand due to the rapid growth of high-quality desktop and mobile videoconferencing solutions.

Based on our results, the magnitude of the carbon reduction *per appointment* is extensive and clearly indicates that up-scaling the use of telemedicine could have a large impact on the over-all carbon footprint of the health sector. The hand- and plastic surgery clinic reduced the carbon emissions per appointment by more than 70 times without having to make major financial investments. The yearly monetary cost of a videoconferencing equipment of this standard is approximately 1100 EUR or 1500 USD. Further, when taking into account trends towards greener transports, telerehabilitation is the most climate-smart work model based on our sensitivity analyses.

These results are from a rural perspective and it is reasonable to expect that the carbon reduction potential of this work model could be significantly smaller in urban environments where patients have shorter distances to travel to receive specialist rehabilitation. However, carbon emissions from a one-hour meeting using a desktop solution are exceeded by the emissions from a car driving as little as a few kilometers based on our baseline assumptions and car queues and traffic jams will significantly increase the emission rates by any type of motor vehicle [23]. Thus, telemedicine also might be promising in cities, particularly those in regions that suffer from poor air quality and experience the majority of all traffic accidents. This new way of thinking about telemedicine and virtual meetings in general could thus be very relevant for city planning and future megacities; reducing risks while saving time and being climate friendly.

When evaluating the potential of telemedicine as climate mitigation strategy, it is important to consider its full potential rather than only assessing the effects from single visits or individual telemedicine programs. Traditional telemedicine activities reduce the carbon costs by only a small fraction in relation to the over all burden of travel, according to a study in the Grampaign region of UK [13]. In this study, telemedicine was estimated to reduce net travel by as little as 0.1%. This brings us to the question whether telemedicine should only be used to serve the needs of those who lack access to health care by virtue of geography, isolation, or other constraints, or if telemedicine can be accepted as an essential component of any ordinary health care activities? For the sake of the environment, we strongly support the latter. To gain true insight into the potential impact of telemedicine on health systems and environment, we anticipate that larger scale studies are needed from the viewpoint of telemedicine as a fully integrated part of any medical or health care activity. Only when becoming part of mainstream medical care and health care can telemedicine reach its full potential as climate mitigation strategy. Some steps in this direction have been taken by the Västerbotten County, which is a geographically widespread and sparsely populated County (population of 260,000) in northern Sweden. Virtual meetings have been highly prioritized for more than a decade and telemedicine is already an integrated part of many clinical units, supporting activities as diverse as surgical planning and follow-ups, specialist rehabilitation, tele-pathology, radiation treatment planning, collaborative care planning and chronic disease management. Consequently, the number of logged videoconferencing hours increased on average 30% per year for the past five years, reaching almost 20,000 hours in 2012 according to the county council statistics. For sake of debate, this corresponds to a reduction of several tons of CO_2_e had all these meetings been applied to clientele and activities similar to the ones addressed in our study. When applying this work model in new clinical contexts it is, however, crucial to take into account possible trade-offs between environmental benefits and clinical outcomes. Intuitively, a clinician is unlikely to adapt a more environmentally friendly solution when there is the potential for any negative impact on the patient or on the quality of services. However, intuitive choices might not accurately reflect actual outcomes. Further, promoting sustainability means the adjustments to current practice should be part of standard evaluation. To support this complex decision process, we suggest continuing this work by performing life cycle impact assessments to estimate the impact on climate change and potential health co-benefits from reductions in CO_2_ emissions.

The method applied in this paper has its limitations; there are very few studies available on this topic and the LCA data on videoconferencing peripherals and transport are sometimes rough estimates and not always completely up to date. It is thus important to keep in mind that the aim of our study was to describe the central aspects of emissions generated by the system under investigation to guide future studies and policy development

There are a lot of global initiatives focusing on reducing the impacts from travel, for example by implementing regulations to reduce tailpipe greenhouse gas emissions, improving vehicle technology, and introducing lower-carbon fuels. At the same time, the rapid development of the ICT sector is leading to more integrated and energy efficient local area networks and less energy demanding PCs and videoconferencing equipments. In contrast to this positive development, the automobile industry is growing steadily, and there are tendencies towards people investing in larger screen sizes and more advanced videoconference systems. These initiatives have the potential to significantly shift the anticipated net reduction in emissions from choosing virtual meeting solutions over face-to-face visits, in either direction. A way to address this in future studies is to adapt methods for calculating time-corrected life cycle emissions intensity for all scenarios by taking into account future development in all relevant sectors, such as ICT and transport [24], [25].

It is a common perception that telemedicine can cut costs, extend health services to remote areas, maintain or improve clinical outcomes, and save time for patients and health workers. Some scientific evidence challenges these perceptions, particularly with respect to cost efficiency [8] but for many telemedicine services, these expectations are unquestionably met [9], [26]. Nonetheless, there is a striking delay in the up scaling and implementation of successful telemedicine work models [27], [28]. There is a lack of studies investigating the impacts of such work models on routine care [29] and a lack of clear organization and economic policies and guidelines [30], [31]; such policies require empirical evidence and dissemination of results to relevant stakeholders and decision makers. It is our hope that the empirical evidence of the environmental benefits of telemedicine generates additional momentum and accelerates the implementation of successful telemedicine systems and work models in health systems globally, for the benefit of patients, health care providers, and the planet.

## Conclusions

This study shows that telemedicine generates far fewer carbon emissions than do traditional care models where patients and health workers are expected to travel by car to appointments. Telemedicine is thus judged to be a potent climate change mitigation strategy, not just in rural areas but also in urban environments where additional co-benefits might be even greater if few people use public or active transport. Although previous research indicates that current telemedicine programs reduce travel-related emissions to a limited extent [14], we conclude that implementing telemedicine more broadly could make a significant contribution to reducing the greenhouse gas emissions driving climate change. This requires a paradigm shift in health care and proactive efforts from health care decision makers. This also calls for empirical evidence on the clinical, economical and environmental benefits of telemedicine. Research is needed on a larger scale to evaluate the current and future impact of different telemedicine solutions on carbon emissions, from the viewpoint of telemedicine as a well-accepted and fully integrated part of any health care activity.
